# Clinical Significance of CK19 Negative Breast Cancer

**DOI:** 10.3390/cancers5010001

**Published:** 2012-12-21

**Authors:** Mamiko Fujisue, Reiki Nishimura, Yasuhiro Okumura, Rumiko Tashima, Yasuyuki Nishiyama, Tomofumi Osako, Yasuo Toyozumi, Nobuyuki Arima

**Affiliations:** 1 Department of Breast and Endocrine Surgery, Kumamoto City Hospital, 1-1-60 Kotoh, Kumamoto City, Kumamoto 862-8505, Japan; 2 Department of Surgery, Kumamoto City Hospital, 1-1-60 Kotoh, Kumamoto City, Kumamoto 862-8505, Japan; 3 Department of Pathology, Kumamoto City Hospital, 1-1-60 Kotoh, Kumamoto City, Kumamoto 862-8505, Japan

**Keywords:** breast cancer, OSNA, CK19, imprint smear cytology, sentinel lymph node, Ki-67, p53

## Abstract

Analysis of sentinel lymph nodes (SLNs) by means of One-Step Nucleic Acid Amplification (OSNA) is gaining widespread use as a quick and accurate method. This assay detects the expression level of cytokeratin 19 (CK19) which is present in some but not all breast tumors. In this study, the clinical significance of negative CK19 was investigated in 219 cases of primary breast cancer. In 179 patients with clinically negative nodes, OSNA and imprint smear cytology of SLN were performed simultaneously. The OSNA revealed a node-positive rate of 24.6%. Negative CK19 correlated significantly with negative ER/PgR and higher Ki-67 values, and marginally with higher nuclear grade and p53 overexpression. The triple negative subtype showed lower CK19 expression. OSNA revealed that one of the negative CK19 cases was actually a false negative but this was corrected with the use of the imprint smear cytology. In conclusion, CK19 negativity reflected the aggressiveness of primary breast cancer. OSNA assay used to analyze SLN was useful, but there is a possibility that it will mistakenly detect false negatives in CK19 negative tumors. Therefore, in tumors with negative CK19, the imprint smear cytology may be more useful in cases with macrometastasis.

## 1. Introduction

Cytokeratin 19 (CK19) belongs to a family of keratins and is a type of cytoskeletal protein that is highly expressed in breast cancer cells [1,2]. CK19 mRNA is a suitable marker for identifying breast cancer deposits in lymph nodes. Therefore, a new semi-automated molecular procedure for rapid intra-operative diagnosis of sentinel lymph node (SLN) metastases in breast cancer patients has been developed using the One Step Nucleic Acid Amplification (OSNA) method. The OSNA-CK19 assay (Sysmex, Kobe, Japan) is based on homogenization of lymph node samples followed by real-time amplification and quantitation of CK19 mRNA directly from the lysate [3]. Pooled analysis of recent studies comparing OSNA with pathology indicated that OSNA is as accurate as pathology (96.3% concordance rate) and is useful for making the decision to omit axillary dissection for OSNA-negative patients (97.4% negative predictive value) [4]. In our institute, this method has been used since June 2010. Imprint smear cytology of several lymph node sections was simultaneously performed as quality control. Our rational for using the imprint smear cytology test is based on previous experience. One of our patients was diagnosed as having a false negative lymph node when we applied the OSNA assay, which caused us to question the accuracy of that procedure. The false negative lymph node was corrected when we used the imprint smear cytology test. Moreover, the primary tumor of the above mentioned case revealed a negative CK19 expression. According to our previous study [5], results of the imprint smear cytology were compared to those of the histological tissue analysis, and imprint smear cytology was equal to the histological analysis in combination with cytokeratin staining. Imprint smear cytology is considered to be useful when the degree of cancer cell atypia and volume of cancer cells in the stump were not low. Therefore, the main section of the primary tumor, focusing on CK19, underwent immunostaining, and correlations were identified between the clinico-pathological features and CK19 expression in the primary breast cancer. Furthermore, the usefulness of imprint smear cytology was evaluated in CK19 negative tumor.

There are several studies that have focused on the CK19 negativity of breast cancer, but the clinical significance of CK19 expression is still unclear. For example, CK19 negativity has been reported as being a poor prognostic factor in young women with triple negative breast cancer [6], and it is correlated with Epithelial-Mesenchimal transition in context of other factors [7]. However, there has been a few reports about relationship between the CK19 expression and clinico-pathological variables such as histological types, biomarkers and more [1,2,6,8]. In this study, we evaluated the clinical significance of CK19 negativity in primary breast cancer.

## 2. Patients and Methods

### 2.1. Patients

The CK19 immunohistochemistry (IHC) of the primary tumors was performed in 219 cases with primary breast cancer from September 2010 to August 2011 at Kumamoto City Hospital, Japan. All the patients received SLN biopsy or axillary nodes dissection. The patients treated with neoadjuvant therapy were excluded from this study. The present study was approved by the ethics committee of Kumamoto City Hospital, and informed consent was obtained from all of the patients.

### 2.2. Histopathological Examination

The factors investigated included the presence or absence of lymph node metastasis, nuclear grade, estrogen receptor (ER)/progesterone receptor (PgR) status, proliferation (Ki-67), human epidermal growth factor receptor-2 (HER2) and p53 overexpression. Immunostaining for ER, PgR, p53, Ki-67 and HER2 was carried out as previously described [9]. The positive cell rates for ER/PgR were determined by IHC, and a value of ≥1% was rated as positive. The proliferative activity was determined by immunostaining for the Ki-67 antibody (Dako, Glostrup, Denmark). The fraction of proliferating cells was based on a count of at least 500 tumor cells. The Ki-67 values were expressed as the percentage of positive cells in each case. In this study, the cases were divided into 3 groups according to our previous study [10]; <20%, <50% and ≤50%. Table 1 shows the distribution of cases as follows; 47.0% in the low group, 39.3% in the intermediate group and 13.7% in the high proliferation group. p53 and HER2 expression was evaluated by immunostaining (labeled streptavidin biotinyated antibody (LSAB) method) with the mouse monoclonal anti-p53 antibody (clone DO7; Dako) and the clone 4B5, Ventana (Tuscan, AZ, USA). The staining pattern of the p53 protein was divided into three groups: 2+ (homogenous and diffuse staining ≥50%), 1+ (heterogeneous or focal staining >5% of cancer cells) and negative (focal staining <5% of cancer cells). p53 (2+) was rated as p53 overexpression. The staining pattern of HER2 was divided into four groups according to the ASCO guidelines [11]: 3+ (strong and diffuse staining >30% cancer cells), 2+ (moderate and diffuse staining), 1+ (focal staining) and negative. Tumors with 3+ staining or 2+ staining and FISH amplification ratio >2.2 were considered positive for HER2.

In terms of CK19 immunostaining, CK19 was assessed by incubating it with the RCK108 primary antibody (Dako) for 20 minutes. Methodology was similar to that performed for ER and PgR, and the the staining (antibody incubation for 20 minutes) for the samples was performed using the VENTANA BENCHMARK XT autostainer. We used I-VIEW DAB Universal Kit (Ventana) for protein detection, which is based on avidin-biotin method.

**Figure 1:**
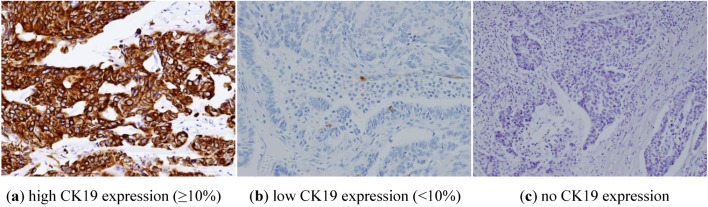
Staining characteristics of CK19 using immunohistochemistry.

CK19 staining intensity was scored as 0 (none), 1 (faint or focal), 2 (moderate), 3 (strong), and percent distribution of cytoplasmic staining. CK19 status was considered positive if the staining intensity was at least 1.0 and had a 10% of epithelial distribution [6]. As seen in Figure 1. The representative cases shows a high expression (a: ≥10%), low expression (b: <10%) and no expression (c) of CK19.

The subtypes were classified as follows according to previously described [12]: ER and/or PgR-positive and HER2-negative breast cancer was classified into luminal A and luminal B (HER2 negative) subtypes using the Ki67 label index. The cutoff point of the Ki67 labeling index was set at 20% according to our previous study [10]; cases with Ki67 of less than 20% were classified as luminal A, and cases with Ki67 greater than or equal to 20% were classified as luminal B (HER2 negative); ER and/or PgR positive and HER2 positive tumors (HER2 IHC: 3+ or 2+ and FISH amplification ratio >2.2) as luminal B (HER2 positive) type; ER and PgR negative and HER2 positive tumors as HER2 positive; and ER and PgR negative and HER2 negative tumors as the triple negative (TN) type.

### 2.3. OSNA

The OSNA assay for CK19 mRNA was first introduced to our pathology department in June of 2010 as a routine method for analyzing of SLN in breast cancer patients. Between September 2010 and August 2011, 179 breast cancer patients from the first series were enrolled in this study to compare the OSNA analysis with the imprint smear cytology of the SLN.

The OSNA protocol consisted of homogenization of tissue in a mRNA-stabilizing solution (Lynorhag, pH 3.5 Sysmex^®^) and subsequent isothermal (65 °C) amplification of cytokeratin 19 (CK19) using the Lynoamp amplification kit (Sysmex^®^) through a reverse transcriptase amplification assay (RT-LAMP) in a gene amplification detector RD-100i (Sysmex^®^) in compliance with the protocol previously described by Tsujimoto *et al*. [3]. Lymph nodes were assessed as negative when there were fewer than 2.5 × 10^2^ copies/μL of CK19 mRNA; positive (1+) when there were between 2.5 × 10^2^ copies/μL and 5.0 × 10^3^ copies/μL; and positive (2+) when there were more than 5.0 × 10^3^ copies/μL [3]. Because some substances interfere with the RT-LAMP reaction, we always prepared the dilute solution of homogenized lymph node. The positive (i+) was determined positive under the inhibition of the RT-LAMP reaction, and CK19 mRNA was 250 copies/μL in the 10-time diluted solution. Positive (1+) was considered as micrometastasis and positive (2+), positive (i+) as macrometastasis [13].

### 2.4. Evaluation of Lymph Node Metastasis Using Imprint Smear Cytology

All nodes that stained blue or those with radioactive counts more than 50 times the background count were defined as SLNs. We omitted axillary dissection in the patients with no metastases in the SLN and performed axillary dissection on the patients with micro- or macrometastases in SLN. In the comparison study, a SLN was sliced into 2 mm thick sections. The technique of imprint smear cytology is as follows. Each fresh cut surface of nodes was imprinted on the slide glass to prepare smear, followed by fixation with 95% ethanol and Papanicolaou staining. After preparation of smear, all sliced tissues were used for the OSNA assay. Cytologists and pathologists diagnosed cancer metastasis using smears, independently of the OSNA assay. We regarded lymph node metastasis as positive when either carcinoma cells were microscopically detected or pancytokeratin, AE1/AE3 was demonstrated positive in atypical cells by IHC.

### 2.5. Statistical Analysis

JMP 8 (SAS Institute Inc., Cary, NC, USA) software was used for the statistical analysis in this study. For statistical processing, the Chi-square test and Fisher’s exact test were used for inter-group comparison (Table 2, Table 3, Table 4). Wilcoxon’s (non-parametric) test was used to compare the mean values for tumor size.

## 3. Results

### 3.1. Evaluation of Lymph Node Metastasis

As shown in Table 1, the age of the patients ranged from 23 to 90 years (mean 58.0), and the mean tumor diameter was 1.6 cm (range 0.1–10). Sixty-four patients (29.2%) were premenopausal and two-thirds (67.1%) of the patients had pathologically negative nodes. In terms of the biological markers, ER- and PgR-positive rates were 77.6 and 71.7%, respectively. HER2 positive rates were 15.5% and the p53 overexpression rate was 14.2%.

One-third (38.4%) of the patients had pathologically positive nodes. Out of the 219 patients, SLNs of 179 cases with clinically negative node were examined by OSNA. The OSNA assay revealed a node-positive rate of 24.6% (44/179) (Table 1). Out of 44 OSNA positive cases, 16 cases (38.6%) had non-SLN metastasis.

**Table 1:** Patients’ characteristics in 219 breast cancer patients.

Characteristics	No. of cases (percentage)
Age (years) Mean ± SD (range)	58.6 ± 13.4 (23–90)
Menopause	
Pre	64 (29.2%)
Post	155 (70.8%)
Nodal status/All cases	
Negative	135 (61.6%)
Positive	84 (38.4%)
OSNA/Cases with SLNB	
Negative	135 (75.4%)
Positive	44 (24.6%)
-Micrometastasis (1+)	30
-Macrometastasis (2+, i+)	14
Number of SLN	
Mean (range)	1.8 (1–5)
Non-SLN metastasis with OSNA positive	
Negative	28 (61.4%)
Positive	16 (38.6%)
imprint cytology/Cases with SLNB	
Negative	150 (83.8%)
Positive	29 (16.2%)
Tumor size (cm)	
Mean (range)	1.64 (0.1–10)
Histological type	
Ductal	188 (85.8%)
Lobular	11 (5.0%)
Others (special type)	20 (9.2%)
-metaplastic carcinoma	3
Nuclear Grade	
Grade 1	46 (22.3%)
Grade 2	146 (66.7%)
Grade 3	27 (11%)
ER status	
Positive	170 (77.6%)
Negative	49 (22.4%)
PgR status	
Positive	157 (71.7%)
Negative	62 (28.3%)
HER2 status	
Positive	34 (15.5%)
Negative	185 (84.5%)
Ki 67	
Median	20.0
Low (<20%)	103 (47.0%)
Intermediate (20%≤, <50%)	86 (39.3%)
High (≥50%)	30 (13.7%)
p53	
Positive (overexpression)	31 (14.2%)
Negative	188 (85.8%)
Subtype	
Total Luminal	170 (77.6%)
-Luminal A	98 (44.7%)
-Luminal B (HER2 negative)	62 (28.3%)
-Luminal B (HER2 positive)	10 (4.6%)
HER2 positive	24 (11.0%)
Triple-negative	25 (11.4%)
Cytokeratin 19 status	
Positive (≥10%)	192 (87.7%)
Negative	27 (12.3%)
-absent	3
-low expression	24

### 3.2. CK19 Status and Clinico-Pathological Characteristics

Table 2 shows the relationships between CK19 status (positive or negative) and clinico-pathological characteristics. CK19 negative tumors were seen in 27 cases (12.3%). Out of these cases, 3 cases showed absence of CK19 expression. CK19 status did not correlate with age, menopausal status, histological type and HER2 status. A negative CK19 significantly correlated with negative ER (*p* = 0.003), negative PgR (*p* = 0.0001) and higher Ki-67 values (*p* = 0.01). Moreover, CK19 expression marginally correlated with nuclear grade (*p* = 0.06) and p53 overexpression (*p* = 0.06). Thus, CK19 negativity reflected the aggressive nature of primary breast cancer.

**Table 2:** CK19 expression status and clinico-pathological factors.

	CK19 negative	CK19 positive	Total	*p*-value
	n = 27	n = 192		
Age (years)				
Mean (range)	58.8 (23–87)	58.4 (30–90)		0.81
Menopause				
Pre	8 (29.6%)	56 (29.2%)	64	
Post	19	136	155	0.96
Tumor size (cm)				
Mean (range)	1.32 (0–40)	1.69 (0–10)		0.36
Nodal status				
Negative	19 (70.4%)	128 (66.7%)	147	0.70
Positive	8	64	72	
Histology				
Ductal	21 (77.8%)	167 (87.0%)	188	
Lobular	1	10	11	0.84
Others	5 ^1^	15 ^2^	20	
Nuclear Grade				
Grade 1	2	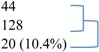	46	
Grade 2	18		146	0.06
Grade 3	7 (25.9%)		27	
ER status				
Positive	15 (55.6%)	155 (80.7%)	170	0.003
Negative	12	37	49	
PgR				
Positive	11 (40.7%)	146 (76.0%)	157	0.0001
Negative	16	46	62	
HER2 status				
Positive	5 (18.5%)	29 (15.1%)	34	0.64
Negative	22	163	185	
Ki 67				
Low (<20%)	13	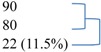	103	
Intermediate (20–50%)	6		86	
High (≥50%)	8 (29.6%)		30	0.01
p53				
Positive	7 (25.9%)	24 (12.5)	31	
Negative	20	168	188	0.06

^1^ Including 1 case with metaplastic carcinoma; ^2^ including two cases with metaplastic carcinoma.

### 3.3. Subtypes and CK19 Expression

Table 3 shows the relationship between subtypes and CK19 status. Luminal type showed higher positive rates of CK19 than TN and HER2 positive. On the other hand, HER2 positive type and TN type showed lower expression of CK19. As shown in Table 1, HER2 status did not correlate with CK19 expression. The incidence of CK19-negative tended to differ between HER2 positive and luminal type, but it was not significant (*p* = 0.07).

**Table 3:** CK19 expression status and subtypes.

	CK19 negative	CK19 positive	*p*-Value	
	n = 27	n = 192	*vs*. Luminal	*vs.* TN
Total Luminal	15 (55.6%)	155 (80.7%)		0.005
-Luminal A	11 (40.7%)	87 (45.3%)		0.03
-Luminal B (HER2 negative)	4 (14.8%)	58 (30.2%)		0.002
-Luminal B (HER2 positive)	0	10 (5.2%)		0.06
HER-2 positive	5 (18.5%)	19 (9.9%)	0.07	0.56
Triple-negative	7 (25.9%)	18 (9.4%)	0.005	

### 3.4. Relationship between OSNA and Imprint Smear Cytology of SLN According to CK19 Status

Table 4 shows the relationship between OSNA and imprint smear cytology of SLN according to CK19 status. In the cases with negative CK19, there was one case that showed a false negative when OSNA was used but could be detected with imprint smear cytology. The other cases that had low CK19 expression showed positive OSNA and also positive imprint smear cytology. In cases with positive CK19 expressions, there were no cases with false negative when OSNA was used. There were 14 (10.6%) cases of false negative when imprint smear cytology was used, and most of them (11/14: 78.6%) had micrometastasis. These findings suggest that it may be difficult to make an accurate diagnosis using imprint smear cytology in cases with micrometastasis. The concordance rate between OSNA and imprint smear cytology was 95% in CK19 negative cases and 91.2% in CK19 positive cases.

**Table 4:** OSNA and imprint smear cytology of SLN according to CK19 expression status.

**CK19 negative**			Imprint smear cytology		Total
			Positive	Negative	
**OSNA**	Positive	Macro	2	0	3 ^1^
		Micro	1		
	Negative		1 ^2^	16	17
			4	16	20
**CK19 positive**			Imprint smear cytology		Total
			Positive	Negative	
**OSNA**	Positive	Macro	25	3	41
		Micro	2	11	
	Negative		0	118	118
			27	132	159

^1^ These three cases that had low CK19 expression showed positive OSNA and also positive imprint smear cytology. ^2^ There was a false-negative case in OSNA.

## 4. Discussion

CK19 is known as an epithelial cell marker and CK19 expression was seen in more than 90% of breast cancer [1,2,3,6,7,8]. According to previous studies, the incidence of tumors with negative CK19 was reported to be 1.4–20%. Alvarenga *et al*. reported that most breast cancer cases were positive for CK19 independent of the histological type. The present study showed the incidence of CK19 negative tumors was 12.3% and expression was absent in 1.4% of the cases. Although there was no significant difference in histological type, CK19 was expressed only in 75% of special type, including metaplastic carcinoma. These findings were consistent with previous studies. There was significantly negative-correlation between metaplastic carcinoma and the presence of CK19. Using CK19 as the sole marker to detect minute foci of breast cancer may result in diagnostic errors.

Tumors with negative CK19 expression significantly correlated with negative ER, negative PgR and higher Ki-67 index value. Moreover, p53 overexpression and higher nuclear grade were often seen in the tumors with negative CK19, although not significant (*p* = 0.06). This data suggests the aggressive nature of CK19 negative tumors. Regarding subtypes, the incidence of CK19 negative expression was higher in TN and HER2 positive subtypes. Zhang *et al*. reported the enhanced expression of CK19 in HER2 positive breast tumors [14]. In this study, the incidence of negative CK19 expression was high in HER2 positive (HER2+ and ER/PgR negative), but HER2 status did not correlate with CK19. Therefore, CK19 status is considered to reflect ER/PgR status more than HER2 status. With respect to TN, similar observation that TN tumors had a higher incidence of CK19 negativity was also made by others [5]. Parikh *et al*. reported that negative CK19 expression showed a significant correlation with negative ER, PR and HER2. They also reported that lack of CK19 expression identifies a subset of patients with a significantly higher risk of local relapse in young women with breast cancer of TN phenotype. Furthermore, it was reported that distant relapse and overall survival rates also correlated with CK19 negativity. TN tumors are usually treated with chemotherapy. ER positive or luminal type tumors are usually treated with endocrine therapy, however, it is still unclear as to the efficacy of treating CK19 negative tumors using chemotherapy even in luminal type tumors. Further research and follow-up is needed.

Regarding OSNA for determination of lymph node metastasis, the false-negative rate in our series was 0.6% (1 case in 179 cases). Therefore, the sensitivity of OSNA was considered acceptable. Although OSNA cannot detect tumor cells with the absence of CK19 expression, tumor cells with low expression of CK19 could be detected in the present study. However, we should consider the possibility of false negatives due to negative CK19, especially in TN and aggressive tumors. Vilardell *et al*. reported that the lack of expression of CK19 is infrequent in breast cancers but also that performing CK19 immunohistochemical staining is important on diagnostic core biopsies when deciding to use OSNA methodology in the evaluation of SLNs in breast cancer patients [15]. According to the present study, imprint smear cytology of SLN was effective in detecting the cancer cells from the CK19 negative tumor. It is difficult to accurately diagnose cancer cells in cases with micrometastasis using imprint smear cytology. Several studies have reported the similar result that the ability of imprint smear cytology to detect micrometastasis is limited [16,17]. However, the misdiagnosis was rare in cases with macrometastasis using imprint smear cytology as mentioned studies indicated, and the correct diagnosis of macrometastasis is meaningful in clinical practice. Therefore, it is important to use imprint smear cytology of SLN simultaneously, especially in CK19 negative cases.

## 5. Conclusions

CK19 expression was often seen in primary breast cancer. The negative CK19 correlated with negative ER, negative PgR, higher Ki-67 values, and marginally with higher nuclear grade and p53 overexpression. Although there were no statistically significance between CK19 negativity and p53 overexpression, higher nuclear grade respectively, trends might exist. These indicate the aggressive nature of primary tumor. SLN analysis by means of the OSNA is a widely used procedure for intraoperative staging of breast cancer patients. In cases with negative CK19, especially the ones that run the risk of appearing as false negatives using OSNA, imprint smear cytology may be more useful in detecting cells in cases with macrometastasis. Further study is needed to evaluate the usefulness of imprint smear cytology.
